# Supplementary material for: Oligodendrocyte precursor cells present antigen and are cytotoxic targets in inflammatory demyelination
Source: Nat Commun. 2019 Aug 29;10:3887. doi: 10.1038/s41467-019-11638-3 (PMC6715717; doi:10.1038/s41467-019-11638-3)
Supplement: Supplementary file 1 — Supplementary Information [file 41467_2019_11638_MOESM1_ESM.pdf]

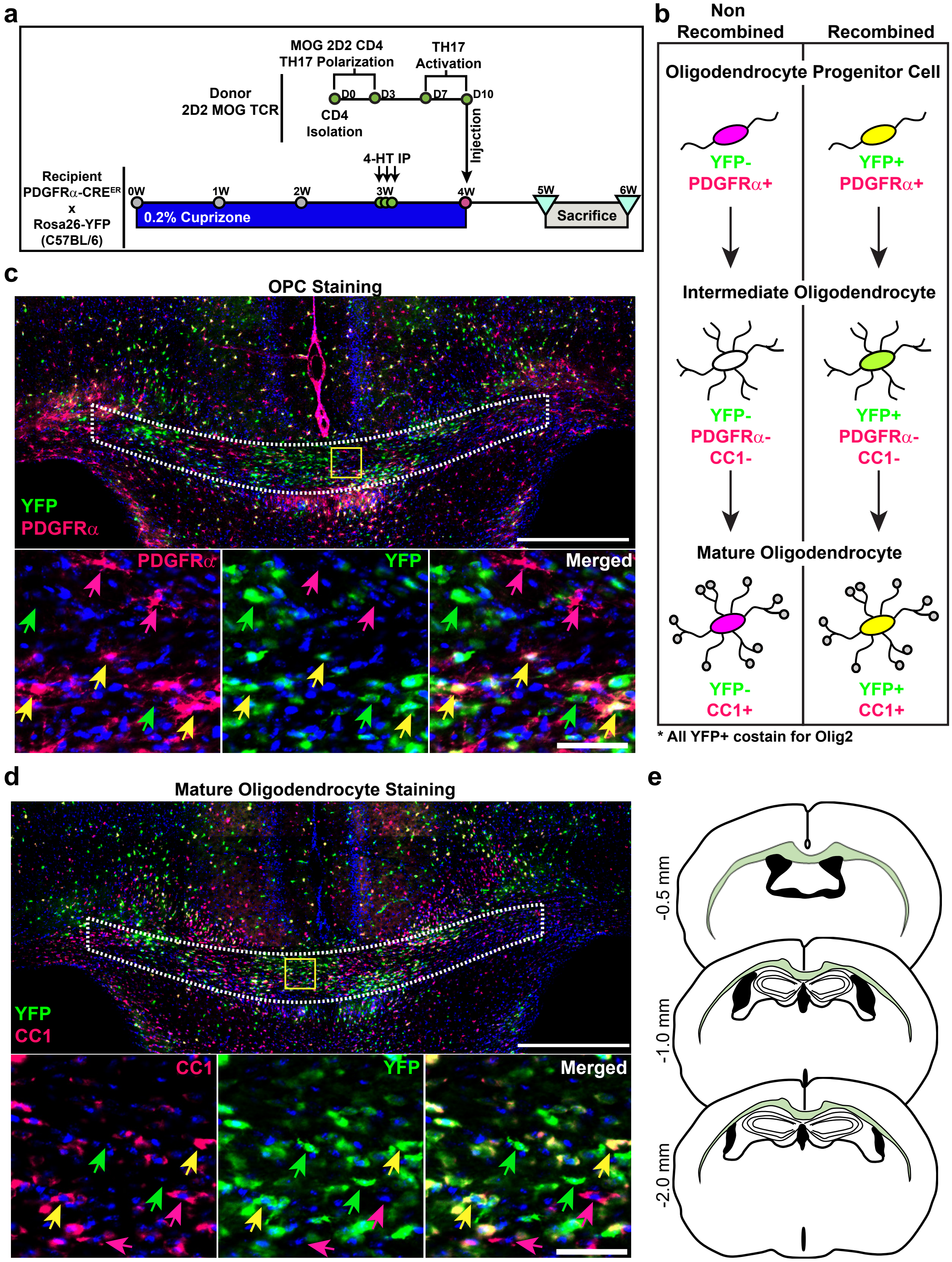

Supplementary Figure 1: Methodology for AT-CPZ oligodendrocyte lineage tracing experiments. (a) Timeline of the experiment for donor and recipient mice. PDGFRα-Cre^{ER} x Rosa26-YFP bred to the C57BL/6 background were kept on a 0.2% CPZ diet for a total of 4 weeks. After 3 weeks CPZ, 4-hydroxytamoxifen (1 mg/mouse/day for 3 days) was injected to induce Cre recombination in PDGFRα expressing cells. Upon recombination PDGFRα expressing cells heritably express YFP regardless of differentiation status. Approximately 8-10 million MOG$_{35\text{-}55}$ specific effector T-cells were isolated and purified from 2D2 TCR transgenic mice and were injected IP into recipient mice at 4 weeks. Simultaneously, the recipient mice were put back on a normal feed diet and were sacrificed 1-2 weeks after adoptive transfer. **(b)** Labeling and quantification strategy for OPCs successfully recombined with 4HT (YFP^{+}) and oligodendrocyte lineage stage identification. Oligodendrocyte lineage cells were grouped into three stages; OPC (PDGFRα^{+}/CC1^{-}), intermediate oligodendrocyte (PDGFRα^{-}/CC1^{-}) and mature oligodendrocyte (PDGFRα^{-}/CC1^{+}). **(c)** OPC staining and quantification (scale bars; 400 μm and 100μm). Recombined OPCs are denoted by the yellow arrowhead, cells stained only for YFP are denoted with the green arrowhead and cells only positive for PDGFRα are denoted by the magenta arrowhead. **(d)** Mature oligodendrocyte staining and quantification (scale bars; 400 μm and 100μm). Mature oligodendrocytes that were recombined at the OPC stage are denoted by the yellow arrowhead, cells stained only for YFP are denoted with the green arrowhead and cells only positive for CC1 are denoted by the magenta arrowhead. **(e)** Bregma locations that were analyzed and averaged for each animal. The corpus callosum highlighted in green was analyzed for each section.

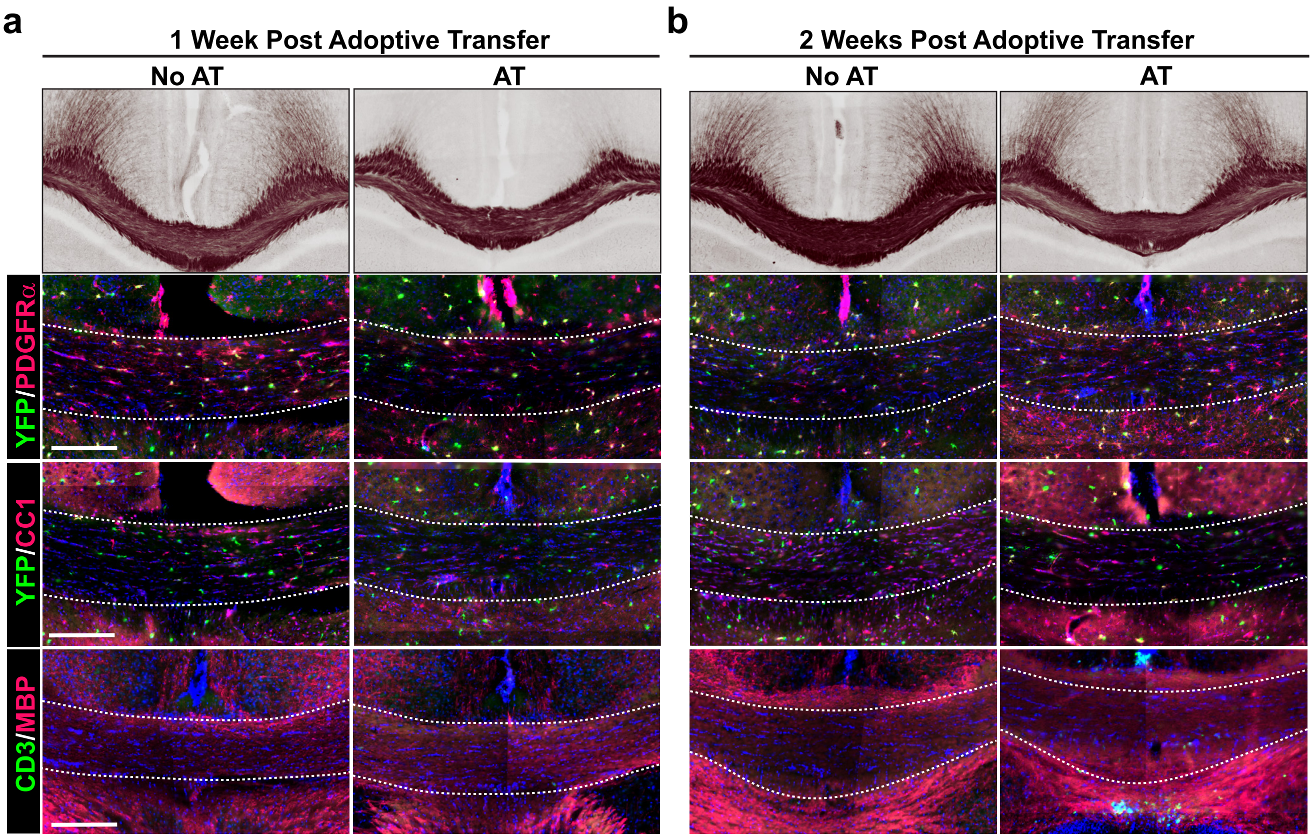

Supplementary Figure 2: Adoptive transfer of effector T-cells inhibits targets the OPC population. PDGFRα-Cre^{ER} x Rosa26-YFP bred to the C57BL/6 background were kept on a 0.2% CPZ diet for a total of 4 weeks. After 3 weeks CPZ, 4-hydroxytamoxifen (1 mg/mouse/day for 3 days) was injected to induce Cre recombination in PDGFRα expressing cells. Upon recombination PDGFRα expressing cells heritably express YFP regardless of differentiation status. Approximately 8-10 million MOG_{35-55} specific effector T-cells were isolated and purified from 2D2 TCR transgenic mice and were injected IP into recipient mice at 4 weeks. Simultaneously, the recipient mice were put back on a normal feed diet and were sacrificed 1-2 weeks after adoptive transfer. **(a)** 1-week **(b)** 2-weeks post AT (scale bar 400 µm) Black Gold myelin staining (**a,b; 1st row**). Representative images of the corpus callosum (outlined by white dashed line) of brain sections. (**a,b; 2nd row**) stained with YFP (green) and PDGFRα (magenta) allowed tracking of recombined OPCs. Representative images of the corpus callosum of brain sections stained with YFP (green) and CC1 (magenta) identified recombined mature oligodendrocytes (**a,b; 3rd row)**. Representative images of the corpus callosum of brain sections stained with CD3 (green) and MBP (magenta) show the distribution of lymphocytes and myelin (**4th row**).

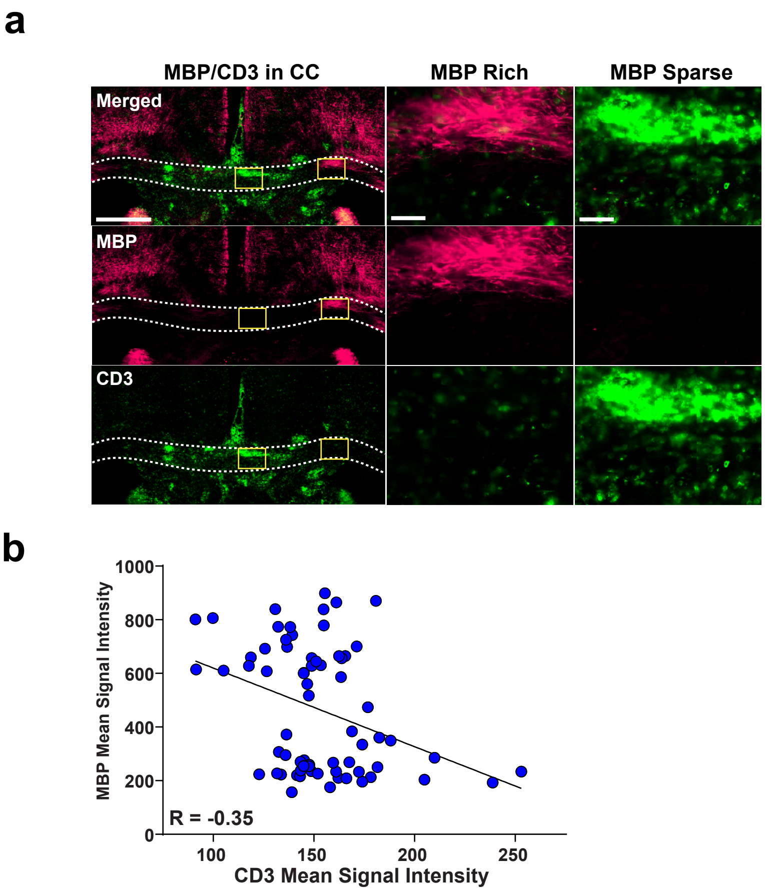

Supplementary Figure 3: MBP and CD3 staining are negatively correlated two weeks after AT into CPZ mice. **(a)** Representative images of the corpus callosum of CPZ + AT mouse stained with CD3 (green) and MBP (magenta) (**left column**) for further analysis of MBP/CD3 distribution (scale bar 400µm and 100 µm). Identification of MBP rich (**middle column**) and MBP sparse (**right column**) regions. **(b)** Linear correlation plot of the signal intensity of CD3 and MBP within the corpus callosum (Pearson Correlation; R = -0.35, P value = 0.003, 95% confidence interval -0.54 to -0.12).

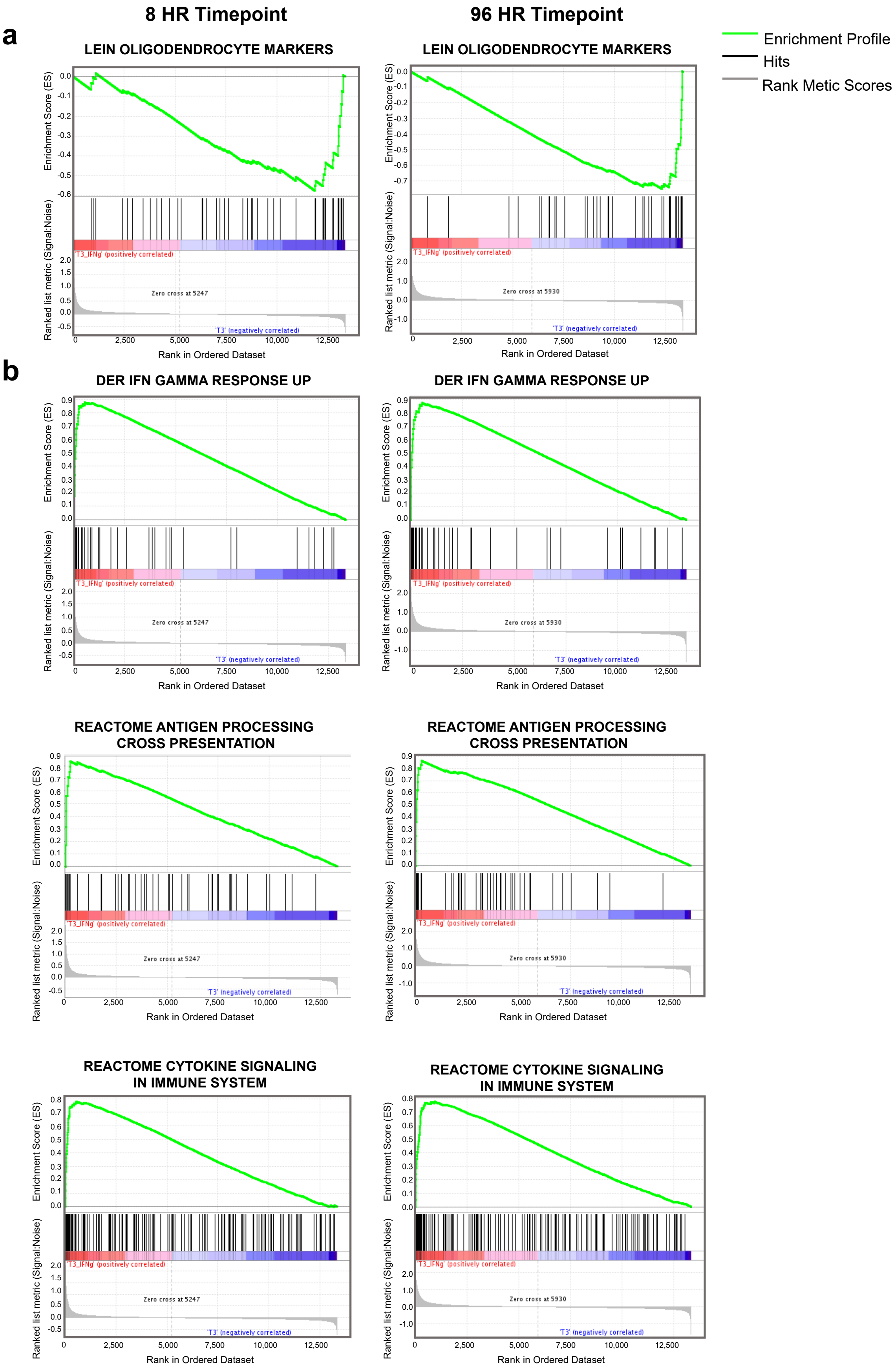

Supplementary Figure 4: Gene set enrichment analysis identifies IFNγ signaling, antigen processing and cross-presentation, and cytokine signaling as major pathways influenced by IFNγ treatment of OPCs. (a) Oligodendrocyte enrichment plot at 8 hrs **(left)** and 96 hrs **(right)**. **(b)** Enrichment plot for IFNγ response **(top)**, antigen processing and cross-presentation **(middle)** and cytokine signaling **(bottom)**, for 8 hrs **(left)** and 96 hrs **(right)**.

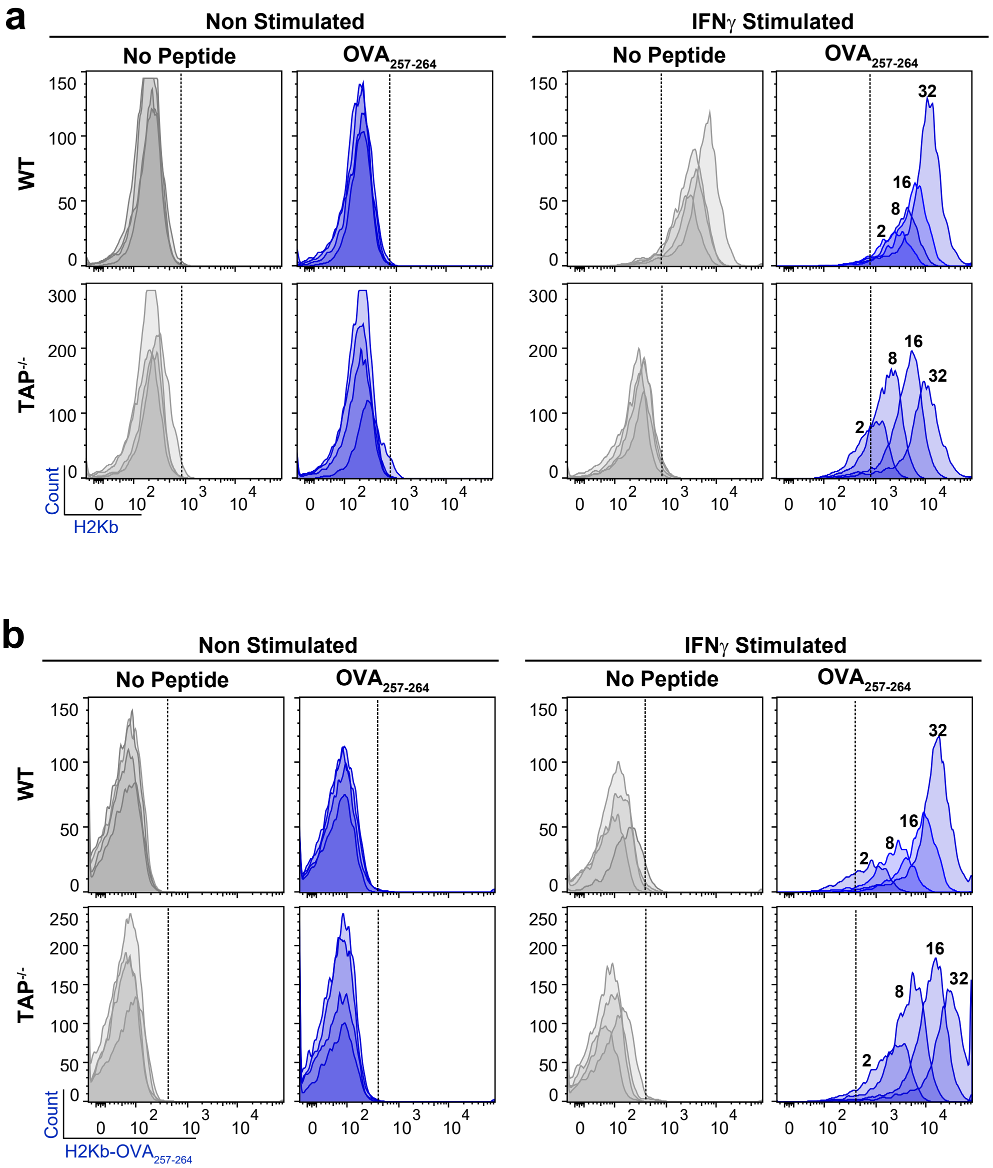

Supplementary Figure 5: IFNγ stimulation induces stable and rapid peptide processing in OPCs. Primary WT C57BL/6 and TAP1$^{-/-}$ mouse OPCs were cultured under PDGF conditions with or without IFNγ (50 ng/mL) for 12 hrs prior to $OVA_{257-264}$ peptide (50 µg/mL) for time course analysis between 0-64 hours. **(a)** Mean fluorescence intensity of total H2Kb expression. (b) Mean fluorescence intensity of $OVA_{257-264}$ loaded H2Kb presentation. H2Kb and $OVA_{257-264}$ loaded H2Kb expression levels increases while even when the percent positive is stable.

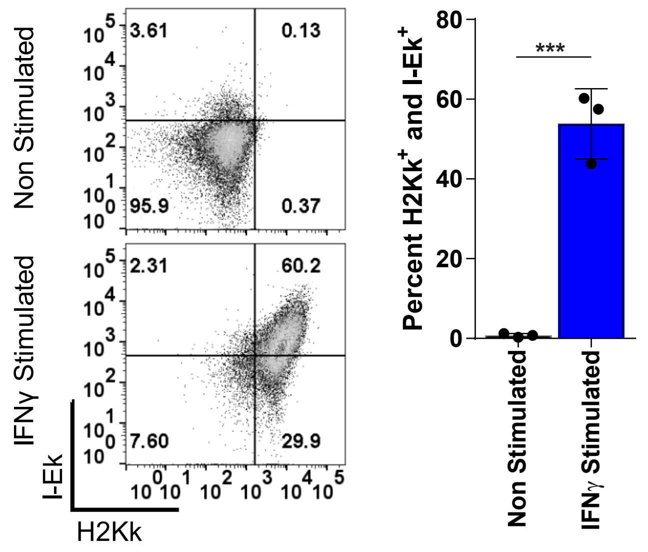

Supplementary Figure 6: IFNγ stimulated OPCs express MHC Class I and MHC class II. Flow cytometry analysis was performed on mouse primary OPC cultures following 24 hours of IFNγ stimulation or no treatment. The OPC population was analyzed by $CD11b^{-}$, $PDGFR\alpha^{+}$ and $A2B5^{+}$ staining. Flow cytometry was quantified, and a significance was determined by two-tailed, unpaired Student's t-test between non-stimulated (gray) and IFNγ (blue) stimulated conditions were compared ($P^* \leq 0.05$, $^{**} \leq 0.01$, $^{***} \leq 0.001$, $^{****} \leq 0.0001$). Error bars represent standard deviation.

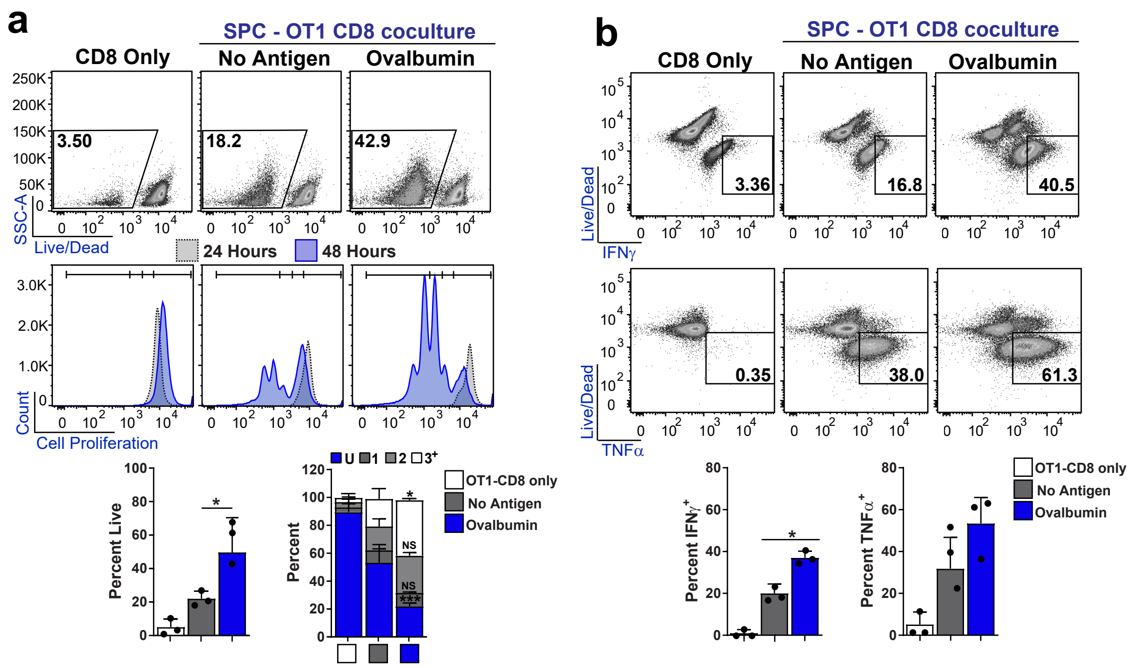

Supplementary Figure 7: Splenocyte activation of OVA-specific CD8⁺ T-cells. Splenocytes were isolated and primed for 8 hours prior to Ovalbumin (500 µg/mL) protein. OT-1 CD8⁺ T-cells were isolated by magnetic sorting then stained with Cell Proliferation Dye eFluor 450 (10 µM) prior to initiation of CD8/Splenocyte co-culture. 24-48 hours after the start of the co-culture CD8 were analyzed for activation. **(a)** Cell survival **(top)** [No Antigen vs Ovalbumin: mean diff = -27.65, 95% CI = -54.25 - -1.060] and proliferation **(bottom:** U; percent undivided, 1,2,3; percent of cells that have undergon 1, 2, or 3 divisions) [(Undivided No Antigen vs. Ovalbumin: mean diff = 37.35, 95% CI = 16.91 - 57.79), (3-Divisions No Antigen vs Ovalbumin: mean diff = -24.85, 95% CI = -45.29 - -4.402)]of OT-1 CD8s alone (white) or co-cultured 24-48 hrs with splenocytes (SPC) under no peptide (gray) or ovalbumin (blue) conditions. Error bars in the quantified figure represents the standard deviation of 3 biological replicates. **(b)** Cytokine and granular protein profiling of OT-1-CD8⁺ T-cells; IFNγ (**1st row**) [mean diff = -17.03, 95% CI = -32.03 - -2.035], TNFα (**2nd row**).

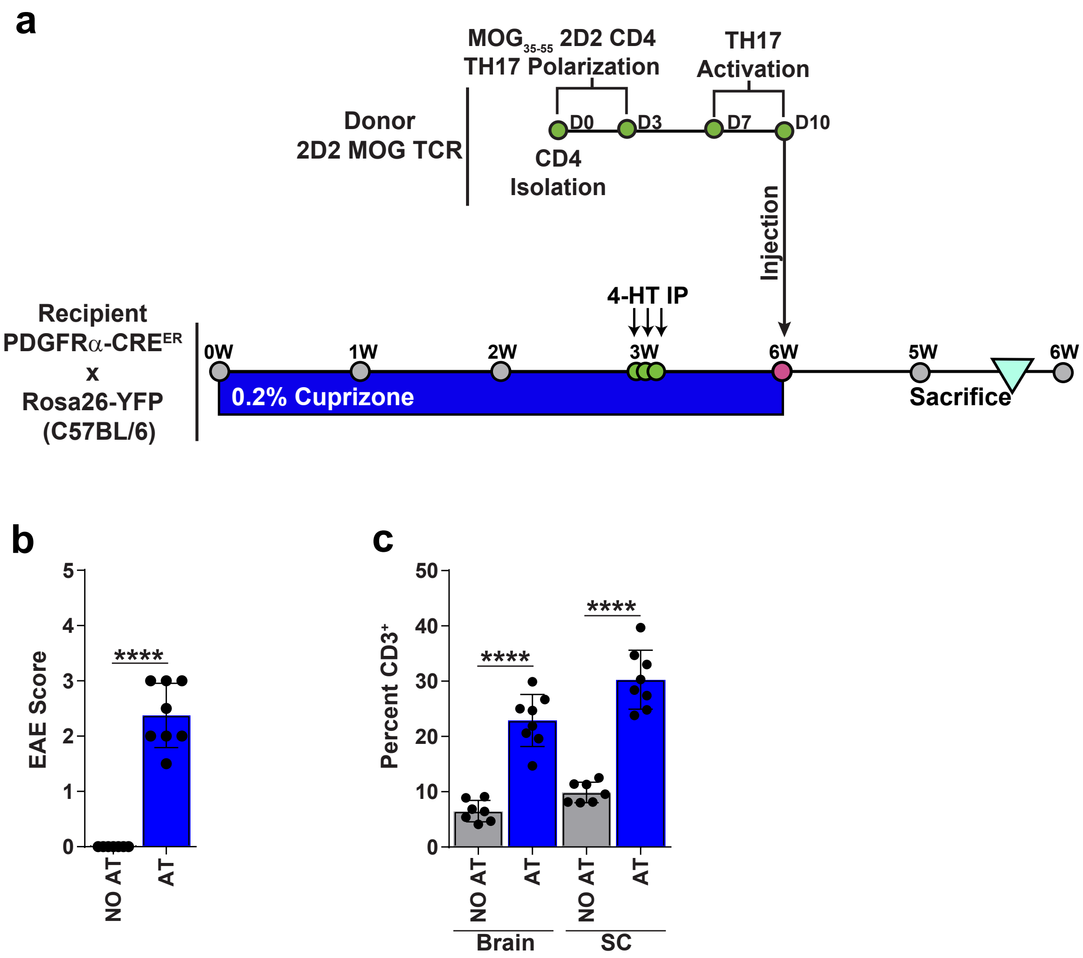

Supplementary Figure 8: Adoptively transferred MOG -specific T-cells infiltrate into the brain and spinal cord. *C57BL/6 PDGFRα-Cre^ER x Rosa26-YFP* were kept on a CPZ diet for a total of 4 weeks. After 3 weeks, 4-HT (1 mg/mouse/day) was injected to induce Cre recombination in PDGFRα expressing cells. Upon recombination PDGFRα expressing cells permanently become YFP^+ regardless of their differentiation status. Approximately 8-10 million MOG_{35-55} specific effector T-cells were isolated and purified using the 2D2 TCR transgenic line and were injected IP into recipient mice after 4 weeks of CPZ feeding. Simultaneously, the recipient mice were put back on a normal feed diet. Twelve days after adoptive transfer when the average EAE score had reached ≥3 the mice were sacrificed and flow cytometry analysis was completed on whole brain tissue. **(a)** The schematic diagram of the in vivo experiment showing donor and recipient mice (described above). **(b)** EAE scores at the time of sacrifice. **(c)** Flow cytometry analysis to determine $CD3^+$ cell infiltration into the brain and spinal cord; CPZ + MOG_{35-55} AT (blue) compared to CPZ alone (gray). All error bars represent standard deviation.

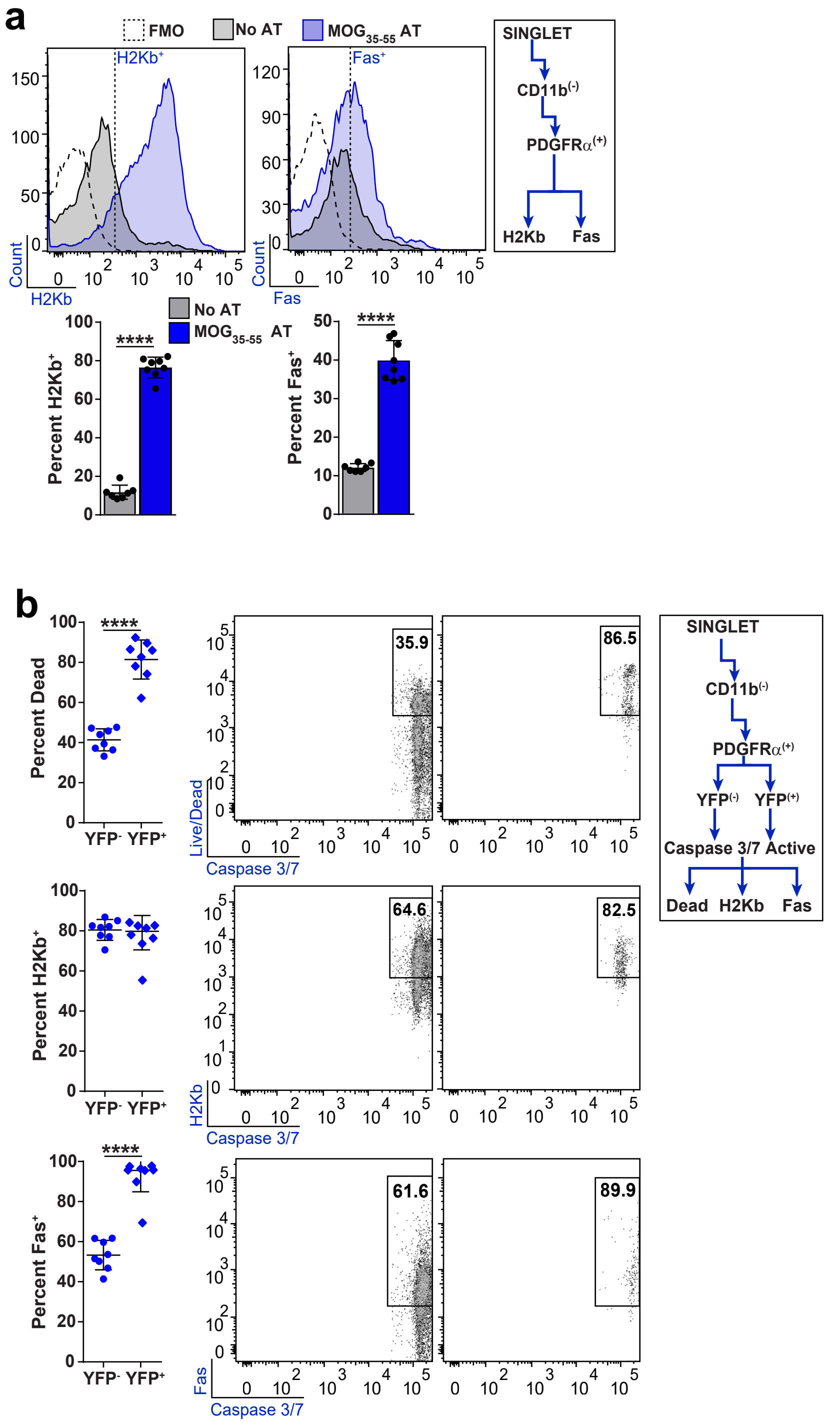

Supplementary Figure 9: OPCs express MHC class I, Fas and are cytotoxic targets in vivo. C57BL/6 *PDGFRα-Cre^{ER} x Rosa26-YFP* were kept on a CPZ diet for 4 weeks. After 3 weeks, 4-HT was injected to induce Cre recombination in PDGFRα expressing cells and MOG$_{35-55}$ reactive T cells were AT. Cells were isolated ex vivo and analyzed by flow cytometry. **(a)** H2Kb **(left)** and Fas **(right)** expression from the OPC population was determined by flow cytometry analysis with quantification below each flow histogram; no AT (**gray;n =7**) and MOG$_{35-55}$ AT (**blue;n=8**). **(b)** Parental gating for the OPC population was distinguished based on YFP$^-$/YFP$^+$ and analyzed for Dead/caspase3/7 active **(top)**, H2Kb$^+$/caspase3/7 active **(middle)** and Fas$^+$/-caspase3/7 active **(bottom)**. YFP$^-$ (**blue circle;n =8**) and YFP$^+$ (**blue diamond;n=7**). Statistical significance was determined by unpaired students T-test. Error bars represent the standard deviation.

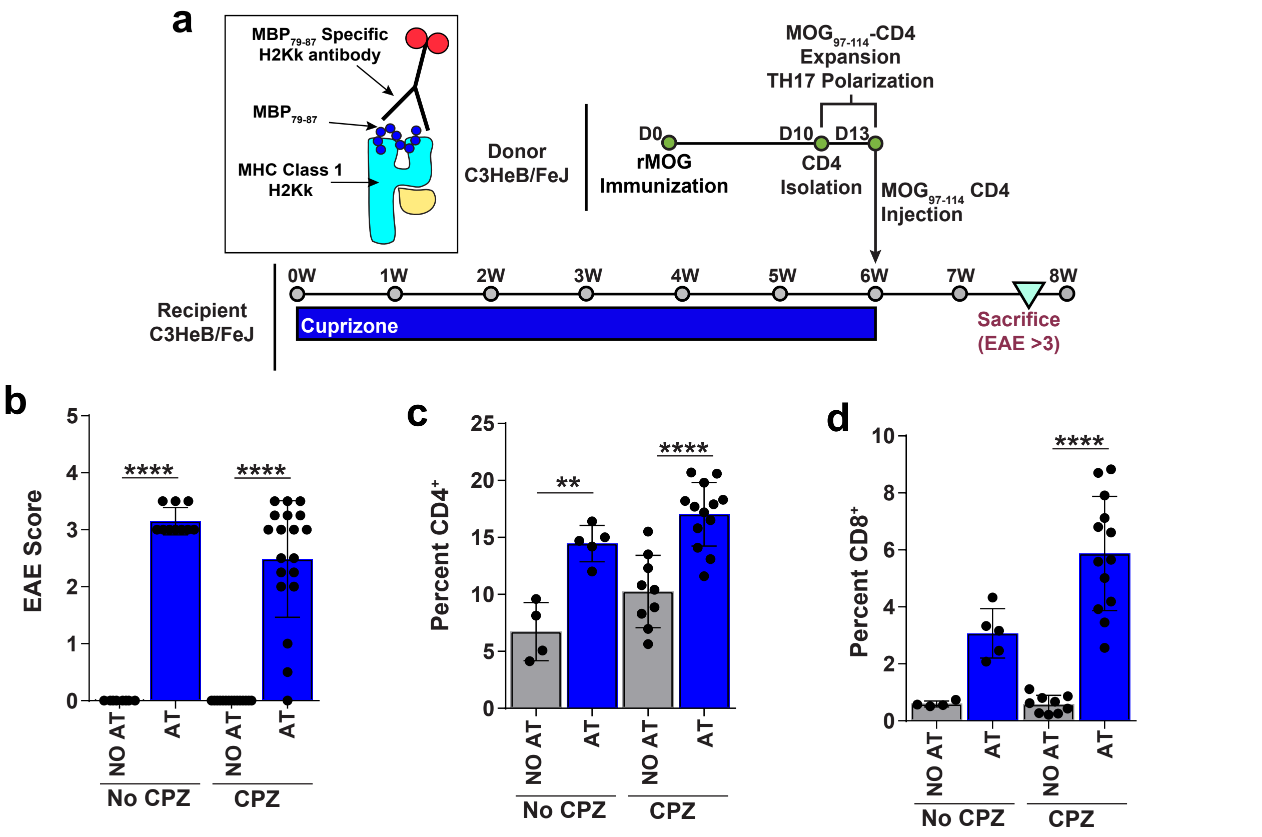

Supplementary Figure 10: Adoptively transferred $MOG_{97\text{-}114}$-specific T-cells infiltrate into the brain and spinal cord. MOG-specific CD4 donor C3HeB/FeJ mice were immunized with recombinant rat $MOG_{1\text{-}125}$ prior to CD4 *ex vivo* isolation and reactivation. Syngeneic recipient mice were fed a CPZ diet for 6 weeks before cultured CD4 cells were adoptively transferred. **(a)** Timeline of experiment (described above) **(b)** EAE scores of experimental mice from three experiments. **(c)** Percentage of brain CD4 infiltration as determined by flow cytometry from a preparation of all CNS cells. **(d)** Percentage of brain CD8 infiltration as determined by flow cytometry from a preparation of all CNS cells. Experimental groups: No CPZ+No AT (**gray; n = 4**), No CPZ + $MOG_{97\text{-}114}$ AT (**blue; n =5**), CPZ + no AT (**gray; n =9**) and CPZ + $MOG_{97\text{-}114}$ AT (**blue; n = 13**). Statistical significance was determined by one-way ANOVA analysis followed by Tukey's multiple comparisons. Error bars represent the standard deviation.

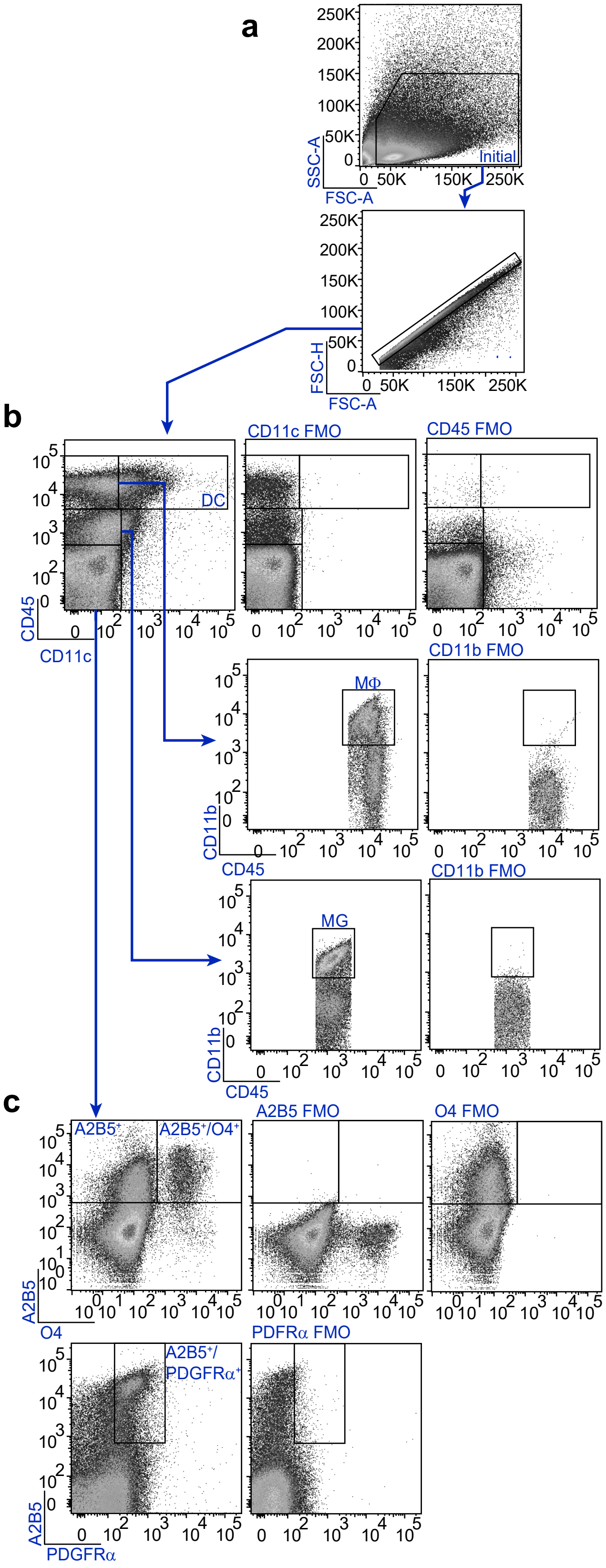

Supplementary Figure 11: Population and gating strategies for CNS flow cytometry. Gating strategy employed for analysis of dendritic cells, microglia, and OPCs. **(a)** Initial **(top)** and singlet gating **(bottom)**. **(b)** dendritic cells, microglia, and macrophages are distinguished based on CD45, CD11c, and CD11b. Fluorescence minus one controls (FMOs) are denoted to show staining specificity. **(c)** The OPC population was determined from the $CD45^-$/$CD11c^-$ population then further gating based on $A2B5^+$, $O4^+$, and $PDGFR\alpha^+$ staining. FMOs are denoted to show staining specificity.
